# Quantitative valve motion assessment in adolescents using point-of-care ultrasound: short communication

**DOI:** 10.1186/s13089-025-00402-y

**Published:** 2025-01-23

**Authors:** Antonio Riera, Lei Chen, Donald S. Wright, Julie I. Leviter

**Affiliations:** 1 Pediatric Emergency Medicine, Yale University School of Medicine, 100 York Street, Suite 1F, New Haven, CT 06511 USA; 2 Emergency Medicine, Yale University School of Medicine, 464 Congress Ave, New Haven, CT 06519 USA

## Abstract

E-point septal separation (EPSS) and tricuspid annular plane systolic excursion (TAPSE) are M-mode measures of left and right ventricular systolic function, with limited pediatric point-of-care ultrasound (POCUS) research. We conducted a cross-sectional study in a pediatric emergency department, enrolling 12–17-year-olds without cardiopulmonary complaints. Exclusion criteria included abnormal vital signs, fever, altered mental status, or psychiatric illness. POCUS faculty performed the measurements, while blinded to pediatric echocardiography reference values. Data was analyzed using unpaired t-tests and Pearson’s correlation. Correlations with age, height, weight, body mass index, and heart rate were examined. Twenty subjects were enrolled. The mean EPSS was 2.5 mm (SD 1.9 mm), and the mean TAPSE was 2.6 cm (SD 0.4 cm), aligning with pediatric echocardiography reference values. No significant correlations were found between EPSS or TAPSE and anthropometric data.

## Introduction

Pediatric visits to community emergency departments constitute a significant portion of children’s emergency care in the United States, often serving as the first point of contact for medical evaluation. Diagnosing critical cardiopulmonary conditions in pediatric patients is particularly challenging due to non-specific presentations and the increased sensitivity of children to ionizing radiation from diagnostic tests. Point-of-care ultrasound (POCUS) is a promising tool for first-line evaluation in this low-prevalence population, particularly for screening critical pathology.

The use of M-mode to assess quantitative measures such as E-point septal separation (EPSS) and tricuspid annular plane systolic excursion (TAPSE) are well-established in adult populations [1, 2]particularly for diagnosing left heart failure and pulmonary embolism [3–6]. In adults, EPSS values greater than 7 mm are strongly associated with severe systolic dysfunction (ejection fraction < 30%), while low TAPSE values (≤ 1.6 cm) are linked to increased mortality risk in pulmonary embolism [7, 8]. However, the pediatric population presents distinct challenges, including lower disease prevalence, variability in clinical presentation, and a paucity of data validating POCUS measures in children.

This study investigates the feasibility of obtaining EPSS and TAPSE values using POCUS in healthy adolescents aged 12–17 years. The primary objective was to compare these measurements with reference standards established by pediatric echocardiography. A secondary aim was to evaluate potential correlations between EPSS and TAPSE values and anthropometric factors, including age, height, weight, body mass index (BMI), and heart rate.

## Methods

We conducted a cross-sectional study in a tertiary care pediatric emergency department, between September 2021 and June 2022. Adolescents were eligible for inclusion if they presented with non-cardiopulmonary conditions, while exclusion criteria included abnormal vital signs, fever, altered mental status, or acute psychiatric illness. POCUS evaluations were performed by POCUS faculty board-certified in pediatric emergency medicine and experienced in cardiac ultrasound, each having performed more than 500 cardiac POCUS on pediatric patients. The senior investigator had completed a POCUS fellowship. Sonologists were blinded to reference values during the study period to reduce potential bias.

**Fig. 1:**
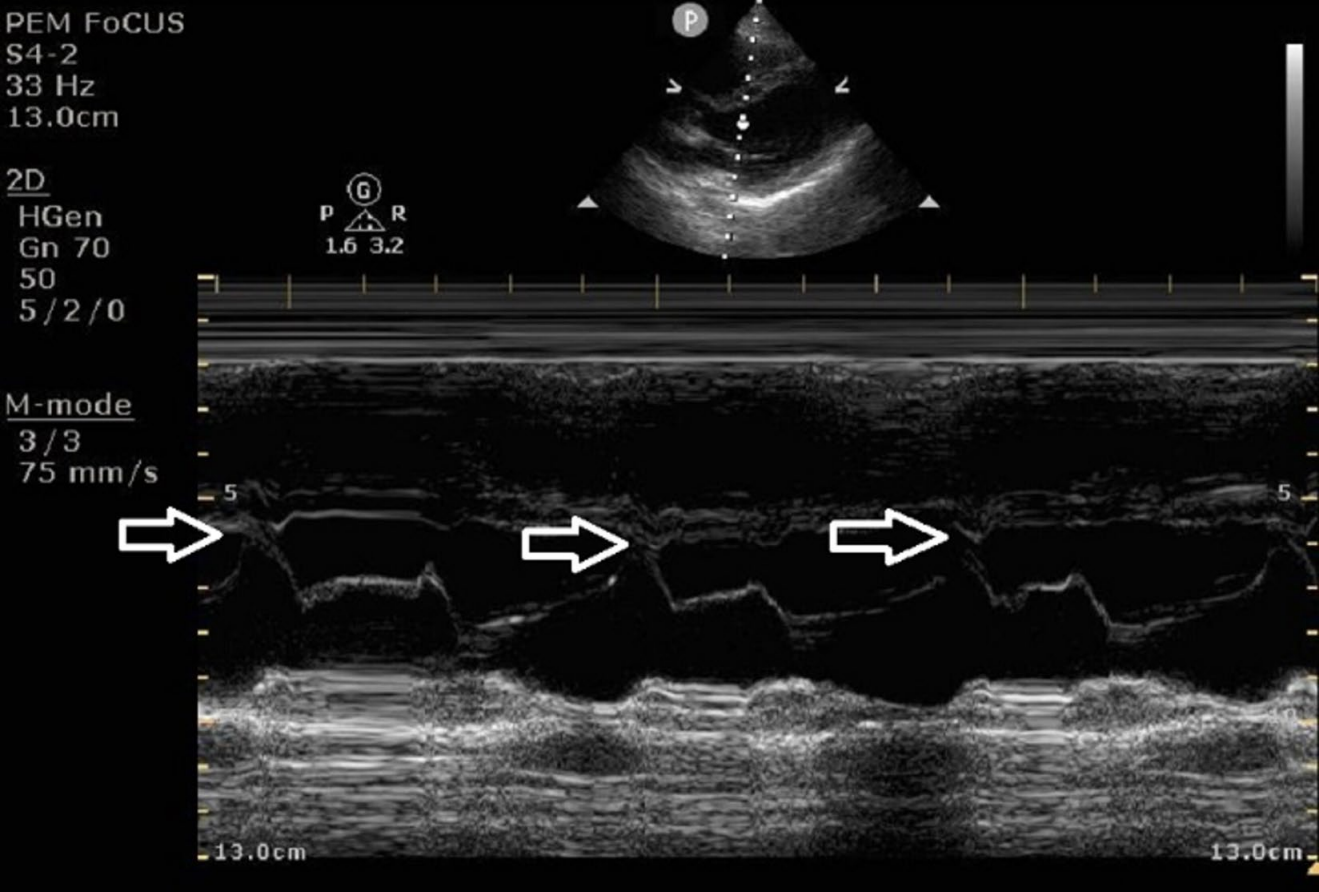
EPSS measurement obtained in the parasternal long axis view. Movement of the anterior leaflet of the mitral valve is seen to contact the septum (arrows) for a measurement of 0 mm

**Fig. 2:**
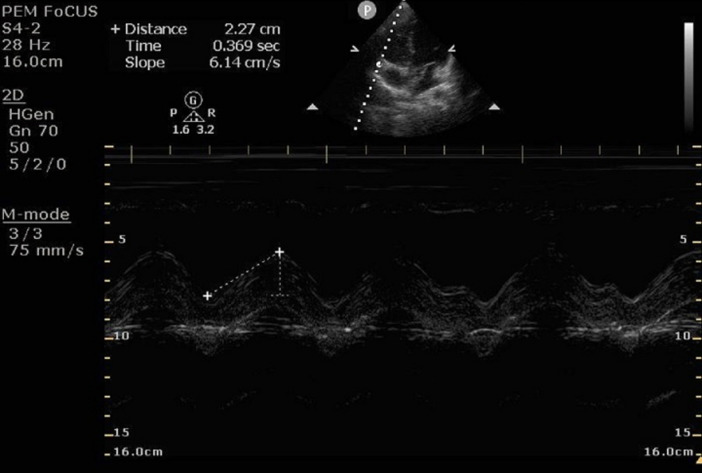
TAPSE measurement obtain in the apical four chamber view. Movement of the lateral tricuspid annulus during the cardiac cycle is measured. The peak to trough measurement is 2.3 cm

Patient height was obtained via verbal report, and weight was recorded from the electronic medical record. EPSS and TAPSE measurements were acquired using a Philips Ultrasound Sparq system. For EPSS, the phased-array probe was positioned in the parasternal long axis view, and M-mode was used to measure the shortest distance between the anterior mitral valve leaflet and the ventricular septum during diastole. A distance of 0 mm was recorded if the leaflet contacted the septum (Fig. 1). For TAPSE, the probe was positioned to obtain an apical four-chamber view, and M-mode was used to measure the displacement of the tricuspid annulus during systole. The tricuspid annulus motion was measured as the distance between the apogee and the lowest point of motion seen on the screen (Fig. 2). Images were saved in a POCUS workflow manager (Qpath, Version 2.24.513 © 2024 Telexy, Inc.) for review as necessary.

Sample size calculations were performed using established means and standard deviations and ensured a 95% confidence interval for the mean measurements. For EPSS, a margin of error of 1.5 mm required a sample size of 15, while for TAPSE, a margin of error of 0.2 cm required a sample size of 19. Unpaired t-tests were used to compare mean EPSS and TAPSE values with pediatric echocardiography reference ranges. Pearson’s correlation coefficient was used to analyze relationships between anthropometric data and M-mode measurements.

## Results

A total of 20 adolescents were enrolled, with a mean age of 14.75 years; 55% were female. The principal investigator enrolled 16 subjects. The other POCUS faculty members enrolled 2 subjects each. POCUS evaluations were successfully performed in all subjects, and no difficulties were encountered in obtaining the required imaging windows or measurements.

The mean EPSS measured by POCUS was 2.5 mm (95% CI: 0.6–4.4 mm). Five patients (25%) had an EPSS of 0 mm, indicating that the mitral valve leaflet contacted the septum during diastole. There was no statistically significant difference between POCUS-derived EPSS values and echocardiography standards (p = 0.42 and 1.0). The mean TAPSE measured by POCUS was 2.6 cm (95% CI: 2.2–3.0 cm). There was no statistically significant difference between POCUS-derived TAPSE values and echocardiography standards (p = 0.91).

While TAPSE demonstrated a weak positive correlation with weight (r = 0.47, p = 0.037), no significant correlations were identified between EPSS and anthropometric variables (Table 1).

**Table 1:** Correlations between EPSS and TAPSE with anthropometric data

	EPSS				TAPSE		
	R value	R^2^	p-value		R value	R^2^	p-value
Age	−0.3477	0.1209	0.134		0.2173	0.0472	0.357
Weight	−0.2263	0.0512	0.338		0.4692	0.2201	0.0369
Height	0.2729	0.0745	0.2443		0.2075	0.0431	0.38
BMI	−0.4148	0.1721	0.0696		0.4323	0.1869	0.057

*BMI* body mass index, *EPSS* end point septal separation, *TAPSE* tricuspid annular plane systolic excursion

## Discussion

This pilot study demonstrates the feasibility of obtaining EPSS and TAPSE values in healthy adolescents using POCUS. The measurements closely aligned with established pediatric echocardiography reference ranges. One study of 84 subjects less than 20 years of age reported a mean EPSS of 3.0 mm (95% CI: 0.4–5.6 mm) and a 0 mm distance visualized 24% of the time [9]. Another study of 105 subjects aged 1 day to 15 years reported a mean EPSS of 2.5 mm (95% CI: of 0.8–4.2 mm), with an upper limit of 6 mm [10]. For TAPSE, a study of 122 subjects between 13–18 years has reported a mean of 2.6 cm (95% CI: 2.2–3.0 cm).

Quantitative POCUS measurements such as EPSS and TAPSE offer an adjunct to traditional qualitative assessments of cardiac function. These measures could enhance diagnostic precision for critical conditions such as heart failure and pulmonary embolism. Importantly, we did not observe any overlap with established measurements in adult patients when severe left ventricular systolic dysfunction (EPSS > 7 mm) or cutoff values shown to be associated with clinically important pulmonary embolus (TAPSE ≤ 1.5 cm, ≤ 1.6 cm, and < 1.8 cm) [8, 11].

The absence of strong correlations between TAPSE and anthropometric data observed in this study contrasts with previous reports of strong associations between valve motion measurements and anthropometric factors. For instance, prior studies have shown positive correlations between EPSS and both height (r = 0.57) and weight (r = 0.60) in broader pediatric age groups [9]. Similarly, TAPSE has been reported to correlate strongly with height and weight (r > 0.8) in large cohorts of children aged 0–18 years [12]. These discrepancies may be attributed to the narrower age range of our study population.

Several limitations warrant consideration. First, the study focused exclusively on healthy adolescents aged 12–17 years, limiting the generalizability of findings to younger children or those with active cardiopulmonary conditions. Additionally, height was self-reported, potentially introducing measurement error. The small sample size precludes definitive conclusions regarding anthropometric correlations and limits the statistical power to detect subtle differences in valve motion measurements. Finally, the evaluations were performed by experienced POCUS faculty, raising questions about the reproducibility of these measurements by less experienced providers. Current recommendations for cardiac POCUS in pediatrics emphasize the importance of training and oversight for qualitative assessments only to evaluate left ventricular systolic dysfunction [13].

While M-mode measurements offer the advantage of reproducible metrics that can track changes over time or be compared to established norms, their reliability may be limited in certain clinical scenarios. Caution should be exercised when conduction delays (e.g., left bundle branch block), structural abnormalities, arrhythmias, or altered hemodynamic states (e.g., severe dehydration) are present, which can alter septal or valve motion. Additionally, obtaining M-mode measurements can add complexity for novice sonologists and may not always be necessary when qualitative assessments provide sufficient information to guide clinical decision-making. Therefore, M-mode measurements should be regarded as an adjunctive tool in pediatric cardiopulmonary assessments, complementing but not replacing broader qualitative evaluations.

Future research should investigate the utility of EPSS and TAPSE in younger pediatric populations and during active disease states such as myocarditis, pulmonary embolism, or asthma exacerbations. Studies should also evaluate the feasibility of teaching these quantitative techniques to trainees and examine inter-observer reliability. Further exploration of the role of quantitative measures by POCUS in differentiating mild versus severe ventricular dysfunction is reasonable. As training and technology evolve, quantitative POCUS measurements have the potential to enhance diagnostic accuracy, reduce reliance on ionizing radiation, and improve outcomes for pediatric patients.

## Conclusion

This pilot study establishes that POCUS-derived EPSS and TAPSE measurements in healthy adolescents are comparable to pediatric echocardiography reference values.

## Data Availability

M-mode results for each subject and their relationship with age, weight, height, body mass index, and the heart rate of our study subjects can be made available upon request.

## References

[1] Navanandan N, Stein J, Mistry RD (2019) Pulmonary embolism in children. Pediatr Emerg Care 35(2):143–151 30702542 10.1097/PEC.0000000000001730

[2] Romer AJ, Rajagopal SK, Kameny RJ (2018) Initial presentation and management of pediatric heart failure. Curr Opin Pediatr 30(3):319–325 29528892 10.1097/MOP.0000000000000624

[3] Satilmis Siliv N, Yamanoglu A, Pinar P, Celebi Yamanoglu NG, Torlak F, Parlak I (2019) Estimation of cardiac systolic function based on mitral valve movements: an accurate bedside tool for emergency physicians in dyspneic patients. J Ultrasound Med 38(4):1027–1038 30265408 10.1002/jum.14791

[4] Daley J, Grotberg J, Pare J, Medoro A, Liu R, Hall MK et al (2017) Emergency physician performed tricuspid annular plane systolic excursion in the evaluation of suspected pulmonary embolism. Am J Emerg Med 35(1):106–111 27793505 10.1016/j.ajem.2016.10.018

[5] Fields JM, Davis J, Girson L, Au A, Potts J, Morgan CJ et al (2017) Transthoracic echocardiography for diagnosing pulmonary embolism: a systematic review and meta-analysis. J Am Soc Echocardiog 30(7):714-U277 10.1016/j.echo.2017.03.004 28495379

[6] Secko MA, Lazar JM, Salciccioli LA, Stone MB (2011) Can junior emergency physicians use E-point septal separation to accurately estimate left ventricular function in acutely dyspneic patients? Acad Emerg Med 18(11):1223–1226 22044429 10.1111/j.1553-2712.2011.01196.x

[7] Lobo JL, Holley A, Tapson V, Moores L, Oribe M, Barron M et al (2014) Prognostic significance of tricuspid annular displacement in normotensive patients with acute symptomatic pulmonary embolism. J Thromb Haemost 12(7):1020–1027 24766779 10.1111/jth.12589

[8] McKaigney CJ, Krantz MJ, La Rocque CL, Hurst ND, Buchanan MS, Kendall JL (2014) E-point septal separation: a bedside tool for emergency physician assessment of left ventricular ejection fraction. Am J Emerg Med 32(6):493–497 24630604 10.1016/j.ajem.2014.01.045

[9] Matzer L, Cortada X, Ferrer P, De Armendi F, Kinney EL (1985) Widened E point septal separation in a normal pediatric population. Chest 87(1):73–75 3965267 10.1378/chest.87.1.73

[10] Engle SJ, DiSessa TG, Perloff JK, Isabel-Jones J, Leighton J, Gross K et al (1983) Mitral valve E point to ventricular septal separation in infants and children. Am J Cardiol 52(8):1084–1087 6637828 10.1016/0002-9149(83)90537-4

[11] Alerhand S, Hickey SM (2020) Tricuspid Annular Plane Systolic Excursion (TAPSE) for Risk Stratification and Prognostication of Patients with Pulmonary Embolism. J Emerg Med 58(3):449–456 31735658 10.1016/j.jemermed.2019.09.017

[12] Uysal F, Bostan OM, Cil E (2016) Determination of reference values for tricuspid annular plane systolic excursion in healthy Turkish children. Anatol J Cardiol 16(5):354–359 26488383 10.5152/akd.2015.6227 PMC5336786

[13] Lu JC, Riley A, Conlon T, Levine JC, Kwan C, Miller-Hance WC et al (2023) Recommendations for cardiac point-of-care ultrasound in children: a report from the american society of echocardiography. J Am Soc Echocardiogr 36(3):265–277 36697294 10.1016/j.echo.2022.11.010

